# Supplementary material for: Co-culture of type I and type II pneumocytes as a model of alveolar epithelium
Source: PLoS One. 2021 Sep 27;16(9):e0248798. doi: 10.1371/journal.pone.0248798 (PMC8475999; doi:10.1371/journal.pone.0248798)
Supplement: S1 File — This word file contains (1) S1 Fig. TEM images of Intracellular void from NCI-H441 culture, S2 Fig. Modelling the relationship between TEER and Lucifer yellow translocation, S3 Fig. TEM image of the tight junction and underlying adherens junction, S4 Fig. TEM images of tight junctions between cells in coculture. (DOCX) [file pone.0248798.s001.docx]

**S1 File**

**This file contains 4 figures**

**S1 Fig. Intracellular void from NCI-H441 culture.**

**S2 Fig. Modelling the relationship between TEER and Lucifer yellow translocation.**

**S3 Fig. TEM image of the tight junction and underlying adherens junction.**

**S4 Fig. TEM images of tight junctions between cells in coculture**


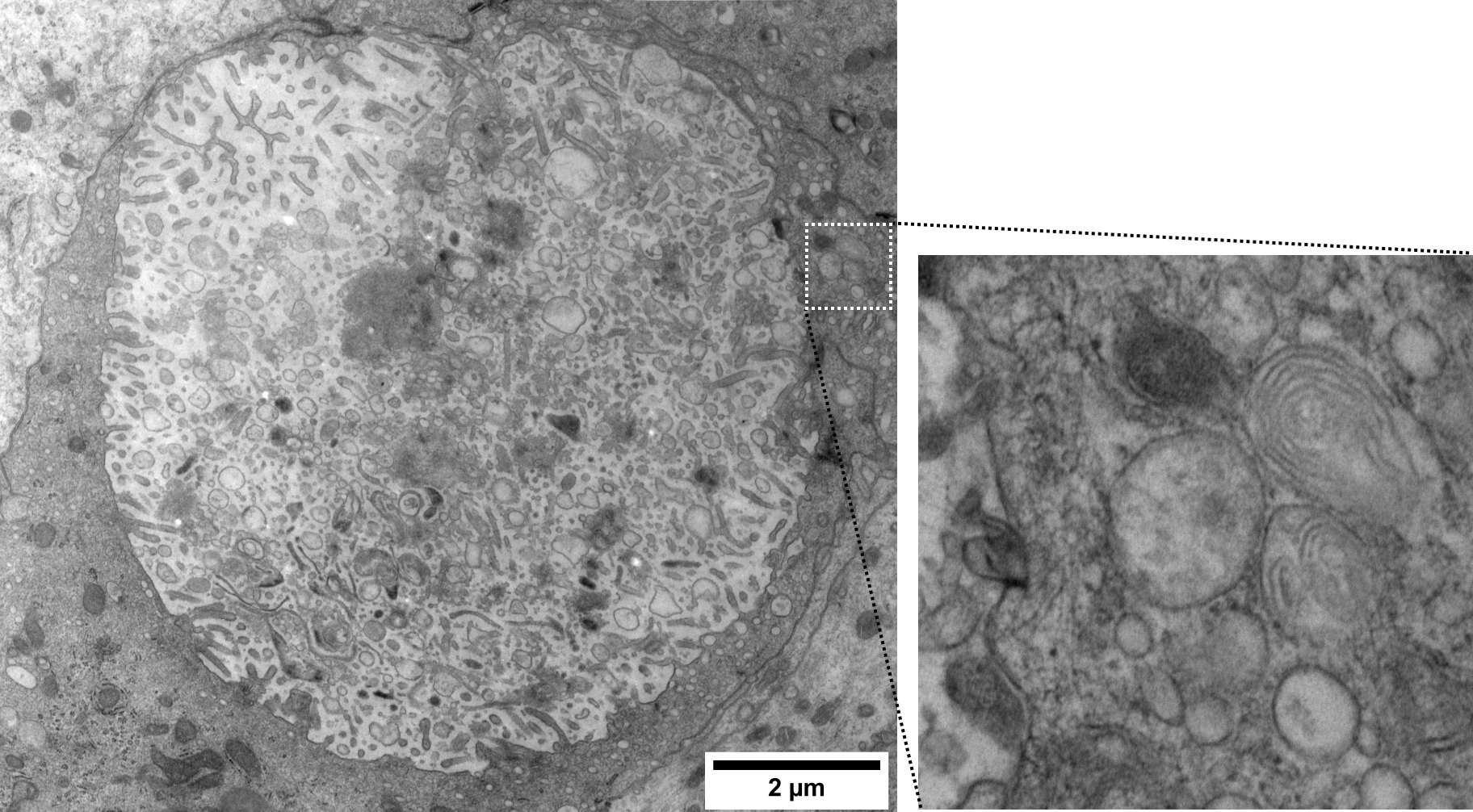


**Fig S1. Intracellular void from NCI-H441 culture** NCIH-441 cells were cultured in ALI conditions for 14 days before being fixed, stained and sectioned for TEM as described in the methods section. This image is a composite of multiple fields to create a complete high-resolution image of one of the intracellular voids found within the cell layer. Several lamellar bodies can be seen in the surrounding cells (inset).


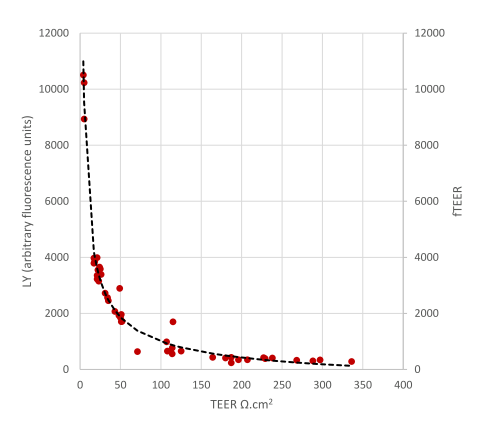


**Fig S2. Modelling the relationship between TEER and Lucifer yellow translocation.** Data were collected from cultures of both hAELVi and NCI-H441 on permeable supports throughout the course of the first two weeks of their development. The graph above shows observed data (red points) of lucifer yellow translocation vs TEER, while the black dotted line shows a function based on the TEER (fTEER) which closely models lucifer yellow clearance. The Solver plugin for excel was used to optimise the offset, scale, and curvature of fTEER to maximise R2 (R2 = 0.98).


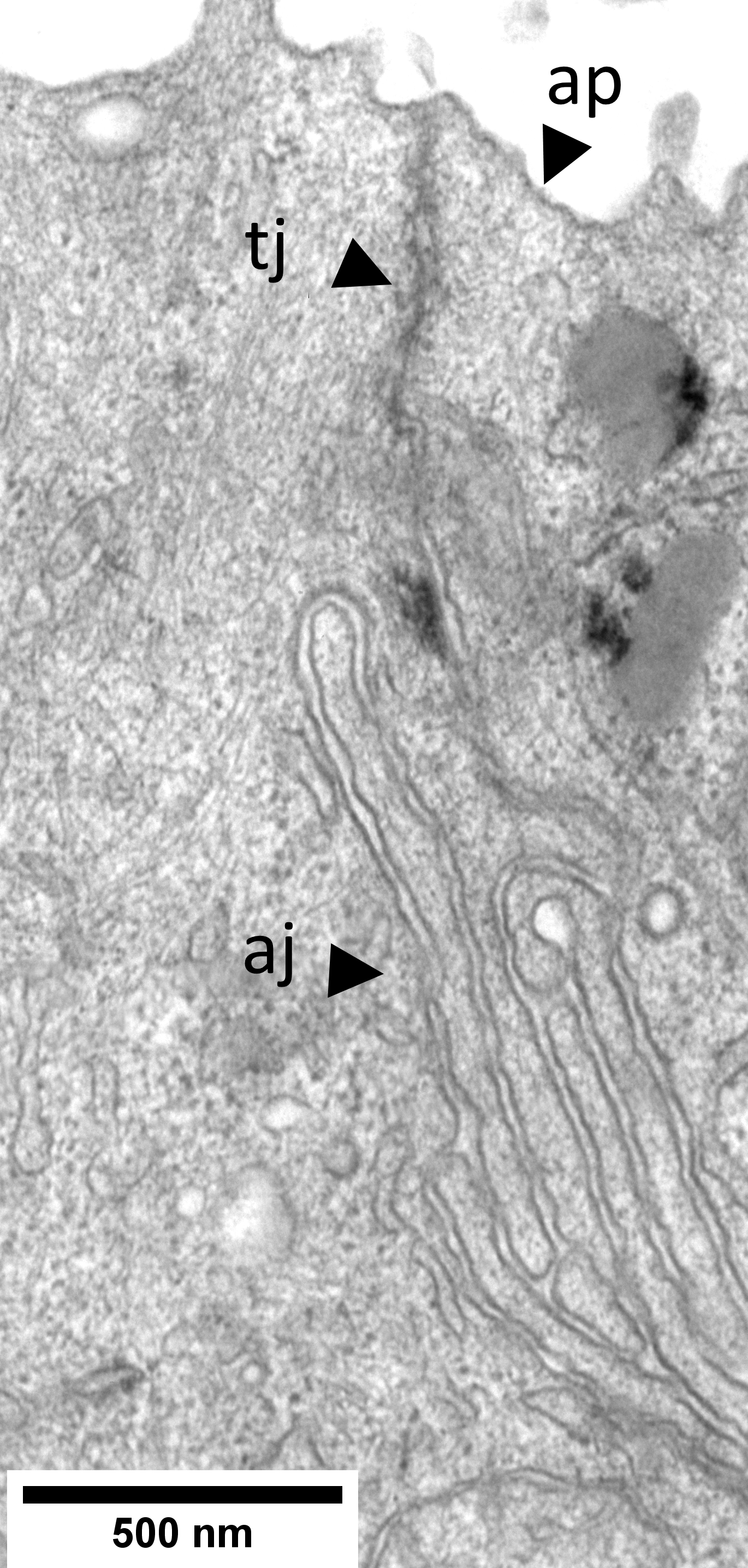


**Fig S3. TEM image of a tight junction and underlying adherens junction in hAELVi cells.** Cultures of hAELVi cells were maintained in ALI conditions for 14 days, fixed, sectioned and stained. Arrowheads demarcate the heavily stained tight junction (tj) lying just beneath the apical surface (ap). Below this it is possible to see the interdigitations of the adherens junction (aj). This image was captured in a slice from a hAELVi single culture, but the same features can be seen in NCI-H441 and in cocultures.


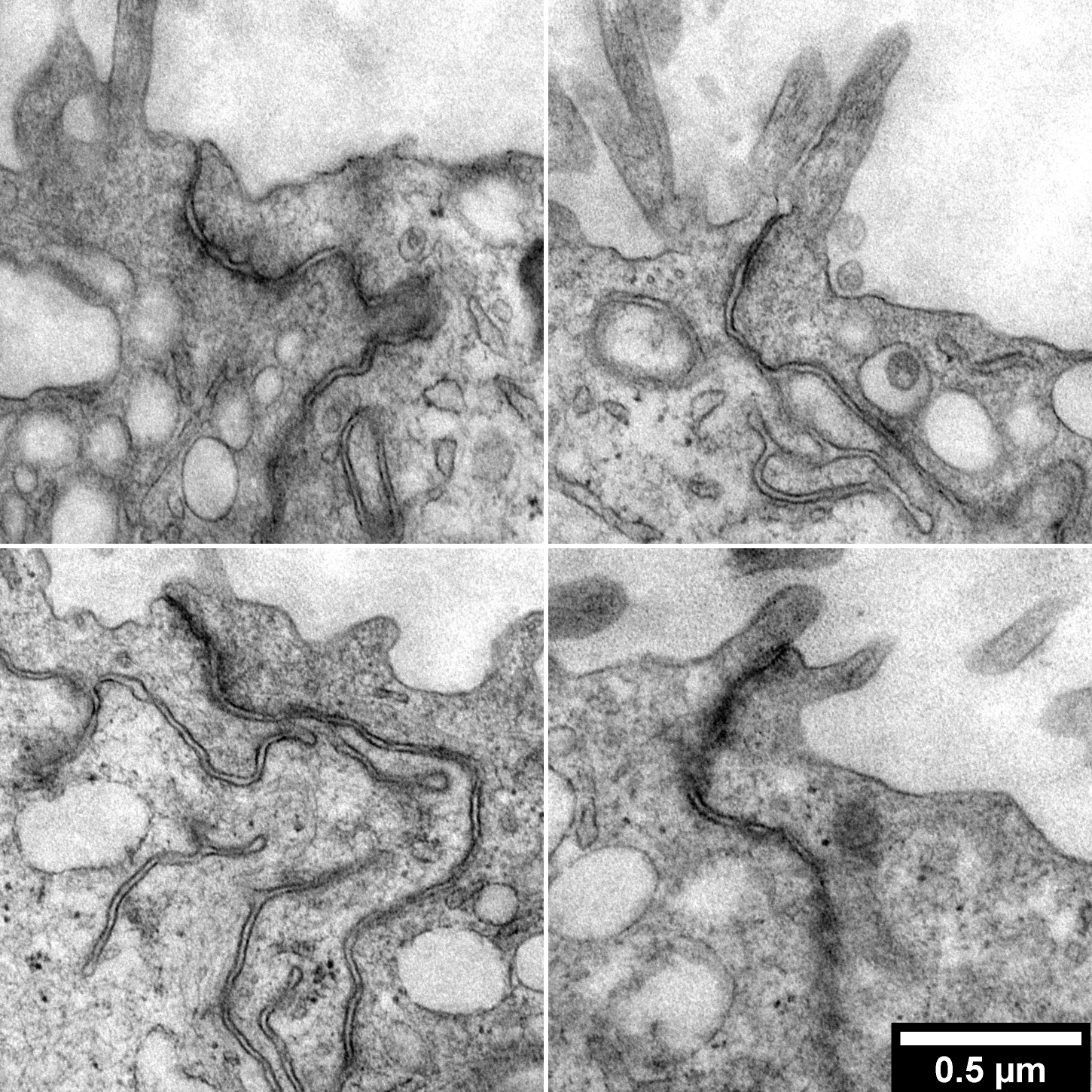


**Figure S4. TEM images of tight junctions between cells in coculture**
