# Supplementary material for: Co-culture of type I and type II pneumocytes as a model of alveolar epithelium
Source: PLoS One. 2021 Sep 27;16(9):e0248798. doi: 10.1371/journal.pone.0248798 (PMC8475999; doi:10.1371/journal.pone.0248798)

Photographs of original western blot membranes.

A- ABCA3 western blot membrane : The ABCA3 data is that reported in Fig 6i. Each gel contains three biological replicates of total protein extracted from ALI interface models based on the individual cell lines or cocultures as marked at the top of the image. Samples were loaded from left to right, starting with the marker as seen in the images. The ladder (M) was PageRuler™ (Thermo), and the image was photographed on an AI6000 digital transilluminator. Experiments were further repeated three times, consistently reproducing these results.

A

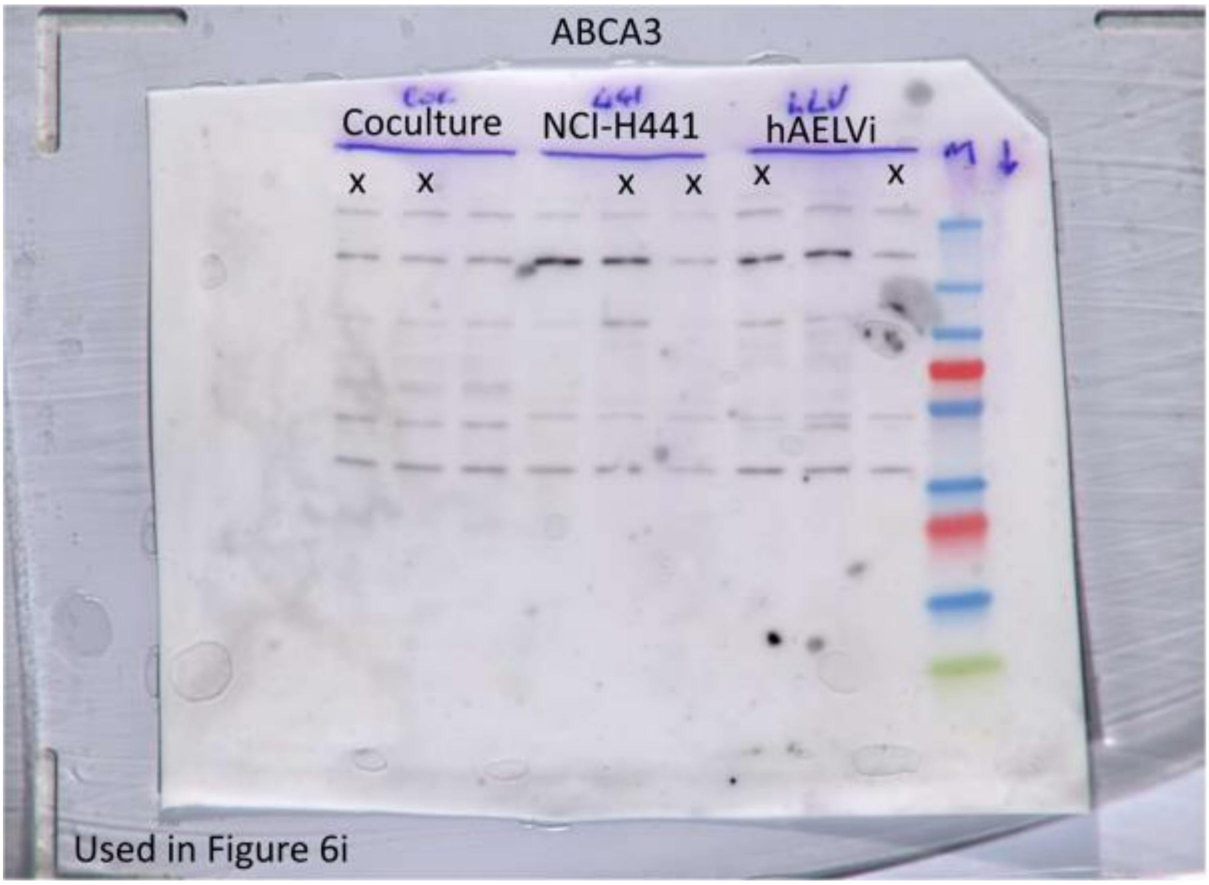

B- Cav-1 western blot membrane : The Cav-1 data is that reported in Fig 7c and d. Each gel contains three biological replicates of total protein extracted from ALI interface models based on the individual cell lines or cocultures as marked at the top of the image. Experiments were further repeated three times, consistently reproducing these results (images a, b, c). Tubulin was used as loading control (images d, e f) to perform quantitative analysis (Fig 7d)

3

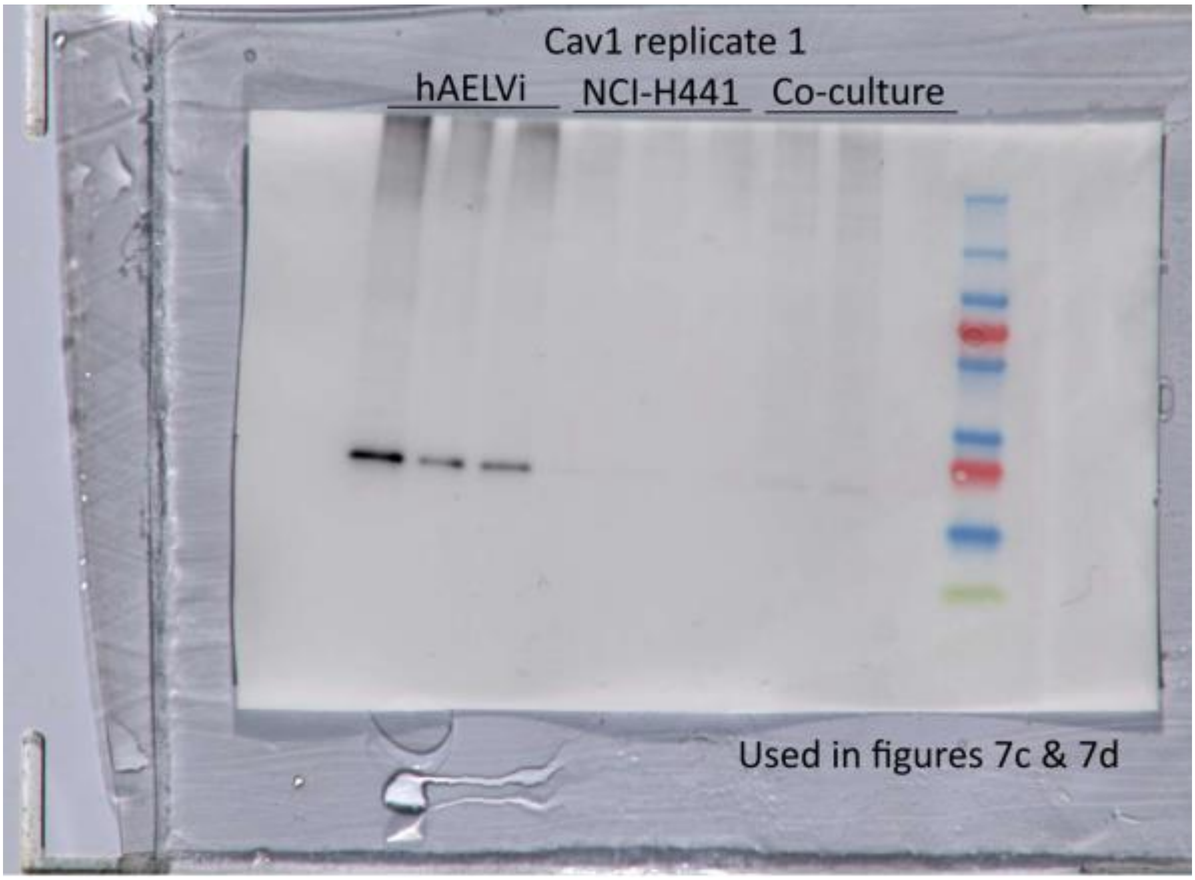

4
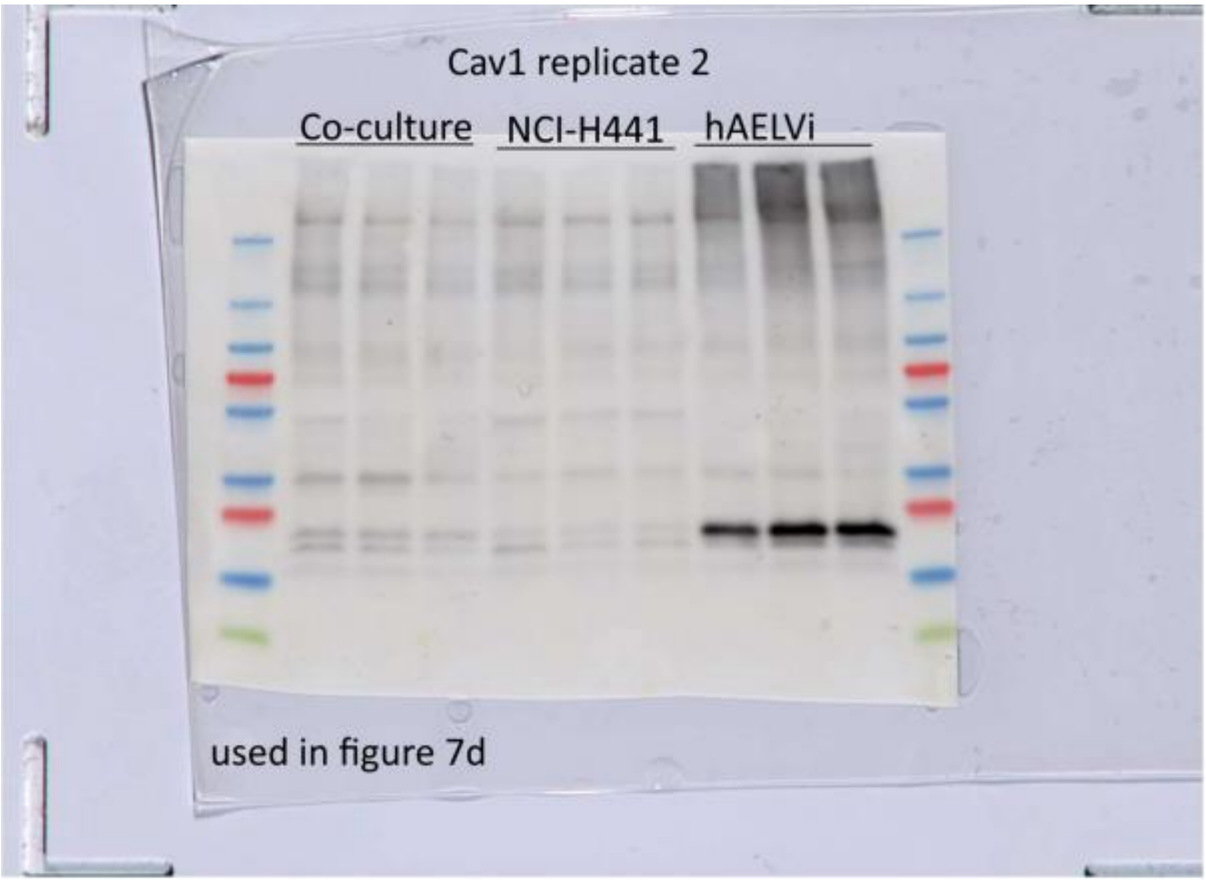
Cav1 replicate 2
Co-culture
NCI-H441
hAELVi
used in figure 7d

5
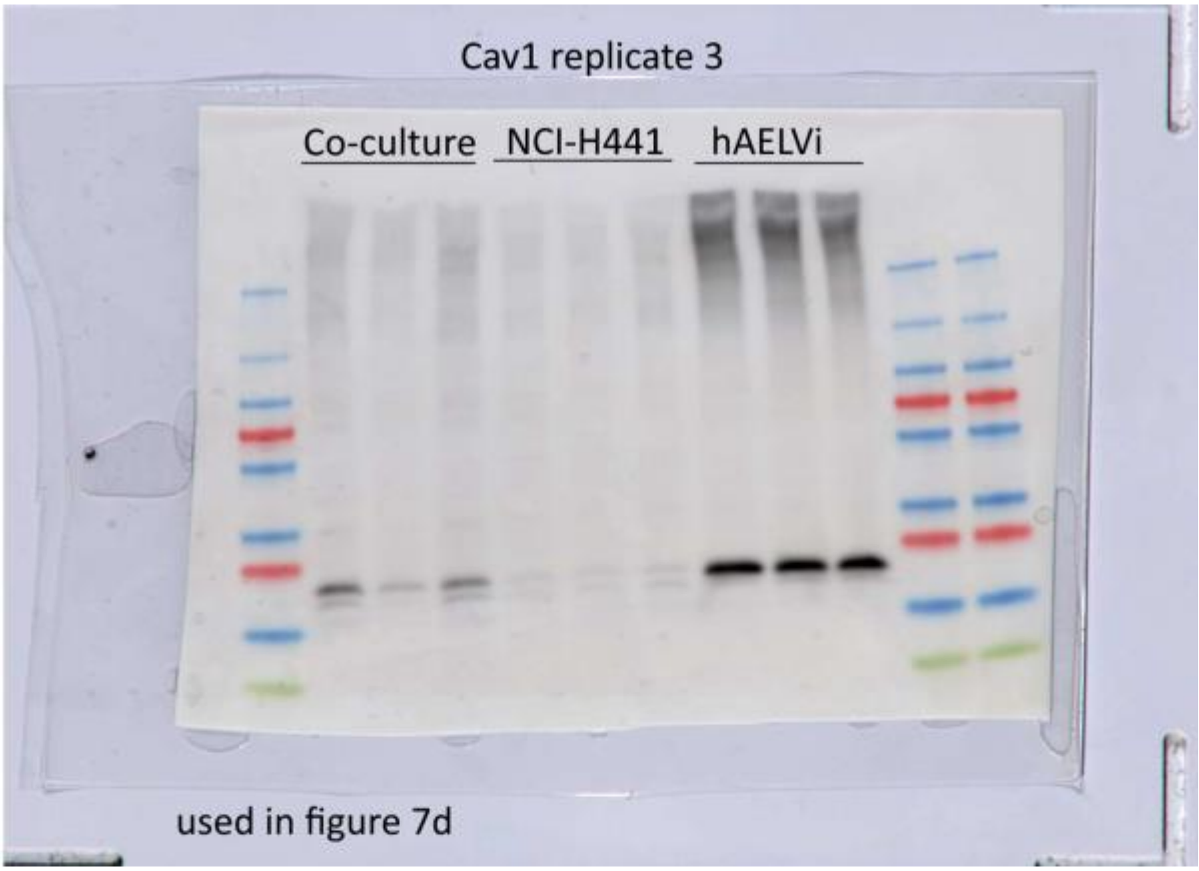
Cav1 replicate 3
Co-culture
NCI-H441
hAELVi
used in figure 7d

d: Tubulin labelling corresponding to blot (a)

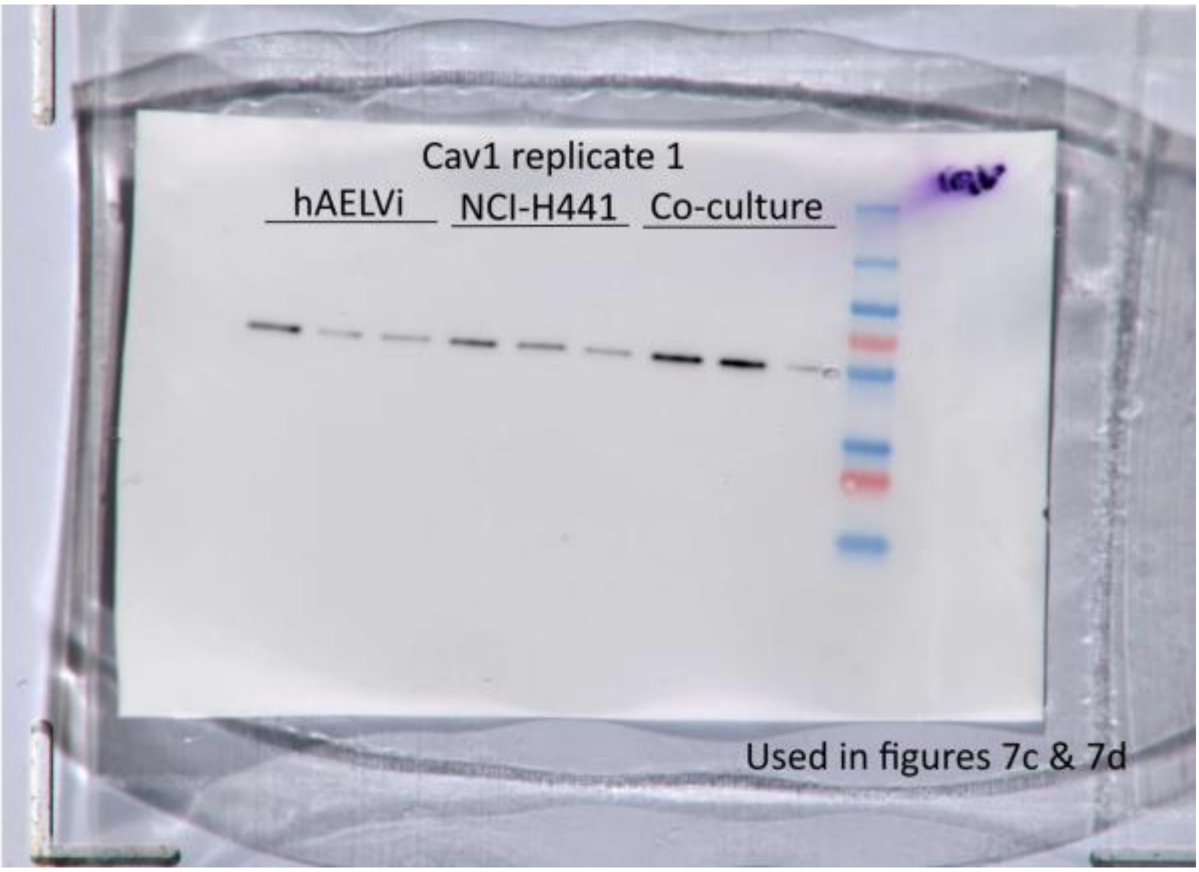

e: Tubulin labelling corresponding to blot (b)

8 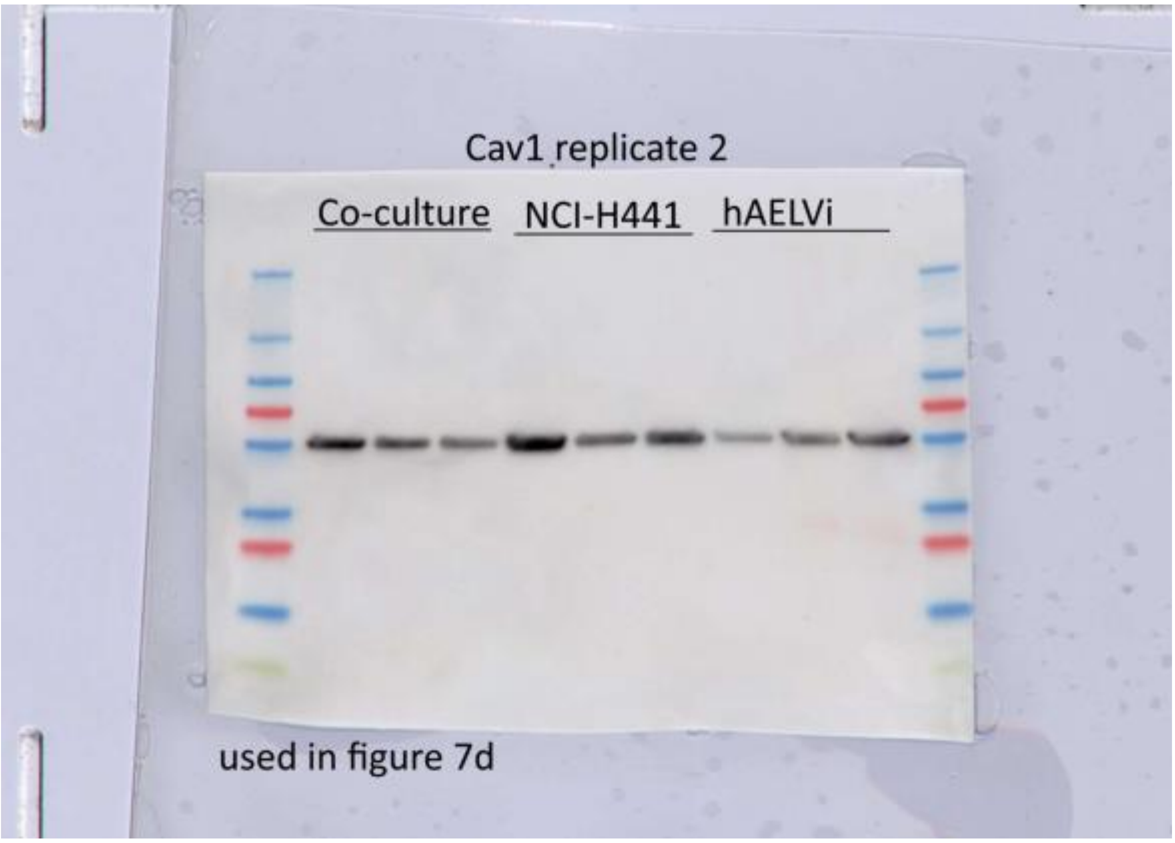

f: Tubulin labelling corresponding to blot (c)

9 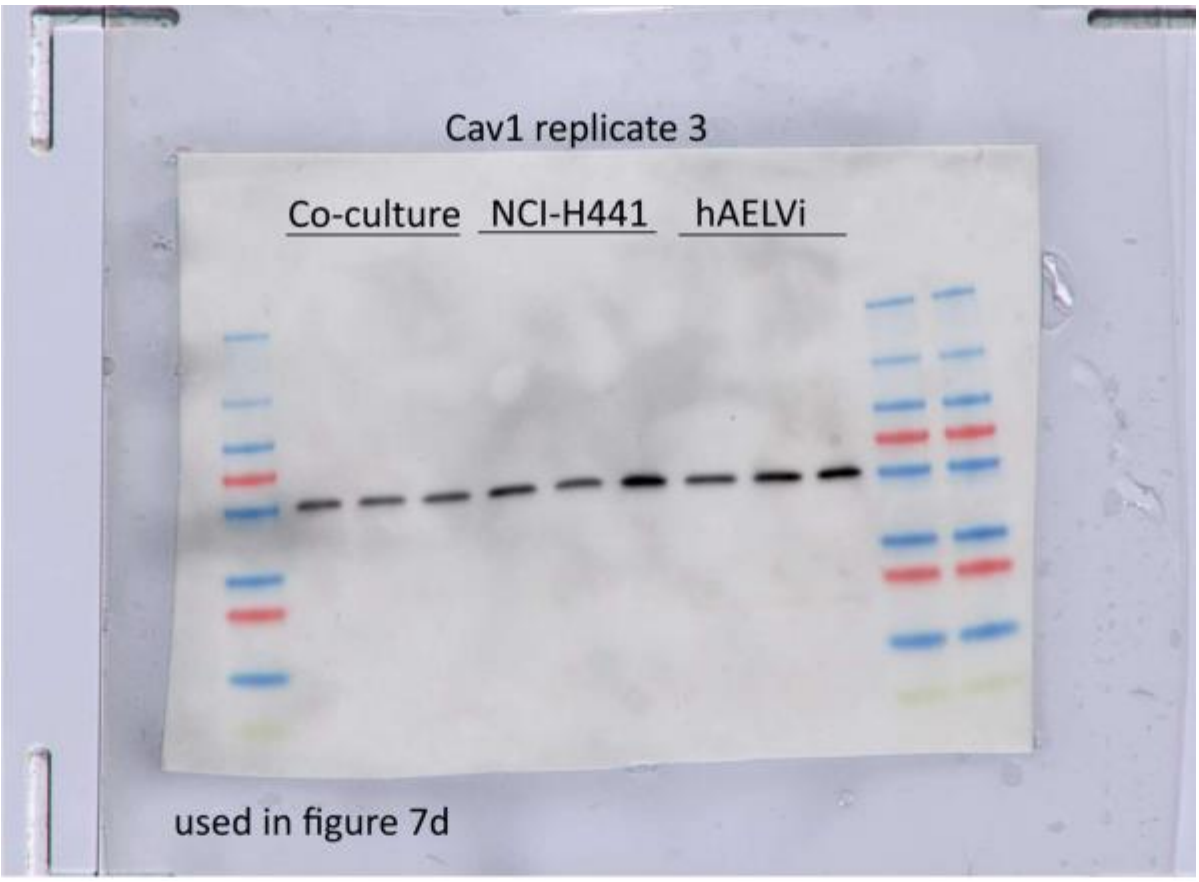

Supplement: S1 Raw images — (PDF) [file pone.0248798.s002.pdf]
